# Characterization of L-type calcium channel activity in atrioventricular nodal myocytes from rats with streptozotocin-induced *Diabetes mellitus*

**DOI:** 10.14814/phy2.12632

**Published:** 2015-11-24

**Authors:** Kathryn H Yuill, Lina T Al Kury, Frank Christopher Howarth

**Affiliations:** 1 Department of Biological, Biomedical and Analytical Sciences, University of the West of EnglandBristol, United Kingdom; 2 College of Sustainability Sciences and Humanities, Zayed UniversityAbu Dhabi, UAE; 3 Department of Physiology, Faculty of Medicine & Health Sciences, United Arab Emirates UniversityPO Box 17666, Al Ain, UAE

**Keywords:** Atrioventricular node, calcium channel, cardiac, diabetes, ion channel

## Abstract

Cardiovascular complications are common in patients with *Diabetes mellitus* (DM). In addition to changes in cardiac muscle inotropy, electrical abnormalities are also commonly observed in these patients. We have previously shown that spontaneous cellular electrical activity is altered in atrioventricular nodal (AVN) myocytes, isolated from the streptozotocin (STZ) rat model of type-1 DM. In this study, utilizing the same model, we have characterized the changes in L-type calcium channel activity in single AVN myocytes. Ionic currents were recorded from AVN myocytes isolated from the hearts of control rats and from those with STZ-induced diabetes. Patch-clamp recordings were used to assess the changes in cellular electrical activity in individual myocytes. Type-1 DM significantly altered the cellular characteristics of L-type calcium current. A reduction in peak *I*_CaL_ density was observed, with no corresponding changes in the activation parameters of the current. L-type calcium channel current also exhibited faster time-dependent inactivation in AVN myocytes from diabetic rats. A negative shift in the voltage dependence of inactivation was also evident, and a slowing of restitution parameters. These findings demonstrate that experimentally induced type-1 DM significantly alters AVN L-type calcium channel cellular electrophysiology. These changes in ion channel activity may contribute to the abnormalities in cardiac electrical function that are associated with high mortality levels in patients with DM.

## Introduction

Cardiovascular complications are common and well documented in patients with type 1 and type 2 DM, leading to an increased risk of mortality (Laing et al. 2003). Disorders of the vasculature, particularly coronary artery disease and hypertension present a two to eightfold increased incidence of mortality in individuals with DM (Malmberg et al. 2001). However, diabetic patients are also at a greater risk of developing cardiac abnormalities, independently of vascular dysfunction, indicative of a distinct diabetic cardiomyopathy (Piccini et al. 2004; Poornima et al. 2006). Evidence from work on experimental models of DM has shown significant impairment of ventricular mechanical function, particularly a reduction in the speed and duration of contraction, and a delay in lusitropy (Bouchard and Bose 1991; Choi et al. 2002). The changes are generally attributed to an alteration in myocyte intracellular calcium handling, and prior studies have observed parallel reductions in the rate of rise and decline of intracellular Ca^2+^ transient activity elicited by electrical stimulation (Choi et al. 2002; Pereira et al. 2006). These events have been attributed to a variety of factors, including altered ryanodine receptor 2 expression and function, sarcoplasmic reticulum (SR) pump activity, SR calcium storage, and altered Na^+^/Ca^2+^ exchange activity (Ganguly et al. 1983; Bouchard and Bose 1991; Schaffer et al. 1997; Yaras et al. 2005).

In addition to altering cardiac contractile properties, DM has a profound effect on the electrical function of the myocardium. Patients have a significantly greater risk of electrical dysfunction, which can manifest as a diverse variety of arrhythmias. QT interval and QRS prolongation are commonly seen and correlate with an increased incidence of sudden cardiac death in diabetic patients (Casis and Echevarria 2004; Movahed 2007; Nakou et al. 2012). Studies have also observed a significant prevalence of atrial fibrillation, in diabetic patients compared to nondiabetic patients (Nichols et al. 2009). Moreover, there is evidence to suggest that AV blocks show greater incidence in diabetic patients compared to the general population (Fairfax and Leatham 1975; Okamoto et al. 1997; Movahed et al. 2005).

Arrhythmogenic activity can result from abnormalities in the conduction properties of the cardiac impulse (Kleber and Rudy 2004), and previous studies using the STZ model of DM have shown that conduction is attenuated in the rat myocardium (Nygren et al. 2007). The changes in conduction may reflect alterations in intercellular gap junctional coupling, manifested by altered expression and activity of connexin proteins 43 and 40 (Howarth et al. 2007a). Moreover, a common substrate for the generation of many arrhythmias, is an imbalance of ionic current fluxes that underlie the cardiac action potential (Marban 2002).

Changes in cardiac ion channel activity occur in animal models of DM, which result in an increased action potential duration (Nobe et al. 1990; Yuill et al. 2010). Previous studies have shown that DM may modify ventricular potassium currents, the transient outward potassium current (Jourdon and Feuvray 1993; Shimoni et al. 1994), the rapid delayed potassium current (Zhang et al. 2006), and the slow delayed rectifier potassium current (Lengyel et al. 2008), thus influencing the repolarization reserve. However, perhaps of greater importance, is the evidence that DM has been shown to modify calcium entry through L-type voltage-dependent calcium channels, the molecular mediators that permit calcium influx required for excitation–contraction coupling. Studies in rabbits, and rats with alloxan, and STZ-induced DM have shown that L-type calcium channel activity is depressed (Wang et al. 1995; Chattou et al. 1999) in ventricular myocytes, and similar findings were observed in a mouse genetic model of type 1 DM (Lu et al. 2007). Furthermore, there is very little information available of the effects of DM on cardiac ion channel function in different cardiac regions, as much of the work to date, has been conducted in ventricular tissue. Studies by Howarth and colleagues (Howarth et al. 2007b) demonstrated that DM can induce changes in sinoatrial node (SAN) conduction and function, during rapid induction of the diabetic state. We have also previously demonstrated that a short-term STZ model of DM can increase action potential duration and modify whole cell membrane conductances recorded unselectively, in rat AVN mycoytes (Yuill et al. 2010). Studies as far back as the 1970s demonstrated the importance of L-type Ca^2+^ channels, which at the time were called slow channels, to AV node conduction (Zipes and Mendez 1973; Zipes and Fischer 1974). L-type calcium channels are fundamental to normal activity in the AVN region (Hancox et al. 1993). They are responsible for mediating the action potential upstroke, and are therefore responsible for the timing of conduction velocity through the AVN, contributing to PR interval duration (Zipes and Mendez 1973; Zipes and Fischer 1974). Modification of L-type Ca^2+^ channel properties either by altered trafficking and expression, or posttranslational modification of channel gating properties, can therefore have a significant impact on AVN function, and result in clinical AV abnormalities. The aim of this study was to characterize the electrophysiological changes induced by DM, using the well-established STZ model on the L-type calcium current (*I*_CaL_) recorded from AVN myocytes.

## Materials and Methods

### Ethical approval

All experiments were performed in accordance with local Ethical Committee approval, United Arab Emirates University, Al Ain.

### Induction of DM

Male Wistar rats received a single intraperitoneal injection of STZ (Sigma, St. Louis, MO) at 60 mg/kg to induce diabetes. STZ was prepared in citrate buffer (sodium citrate/citric acid, 10 mmol/L, pH 4.5) immediately before administration. The induction of a diabetic state was confirmed prior to experimentation by an elevated blood glucose concentration of 440.9 ± 12.3 mg/dL compared to control levels of 97.7 ± 6.2 mg/dL, measured using a glucometer. All animals were maintained for a minimum of 12 weeks on the same diet and water. Age-matched male rats (302.6 ± 9.6 g) were used as control animals. STZ rats had a significantly smaller body mass, 239.2 ± 6.3 g.

### Atrioventricular myocyte isolation

Control (*n* = 14) and STZ-treated diabetic rats (*n* = 14) were killed by cervical dislocation and decapitated. The heart was rapidly removed from the chest cavity, and placed in ice-cold isolation solution prior to aortic cannulation, and subsequent retrograde Langendorff perfusion (6 mL/min/g heart tissue). The hearts from control animals had a mean weight of 1.17 ± 0.04 g and those from STZ animals weighed 0.96 ± 0.02 g. In the first instance, the hearts were perfused for 2 min with isolation solution containing the following constituents (in mmol/L): NaCl (130), Hepes (10), glucose (5), taurine (20), creatine (5), KCl (4.5), NaH_2_PO_4_ (1), Na-pyruvate (5), MgCl_2_ (5), and CaCl_2_ (0.75 *μ*mol/L) (pH adjusted to 7.25 with NaOH, and bubbled with 100% O_2_). The hearts were then perfused with calcium-’free’ isolation solution, containing 8 *μ*mol/L Na-EGTA, before switching to isolation solution containing CaCl_2_ (150 *μ*mol/L), collagenase type 2 (1 mg/mL, Worthington), protease type X1V (0.1 mg/mL, Sigma), and bovine serum albumin (1%, Sigma) for 6–10 min. Hearts were then removed from the cannula, the AVN region was identified and excised, and AVN myocytes were isolated by enzymatic and mechanical dispersion (Hancox et al. 1993; Yuill et al. 2010). After isolation, cells were stored in a high [K^+^], low [Ca^2+^] storage solution at 4^°^C for use on the same day. This contained (mmol/L) l-glutamate (110), KCl (30), Na-pyruvate (5); Taurine (20), creatine (5), succinic acid (5), Na_2_ATP (2), *β*-hydroxybutyrate (5), glucose (9); MgCl_2_ (5), EGTA (0.05), HEPES (10), phosphocreatine (1), K_2_HPO_4,_ (5), and had a pH of 7.2.

## Electrophysiological recordings

Isolated AVN myocytes were placed in a recording chamber, mounted on an inverted microscope. They were allowed to adhere to the glass base of the chamber, before commencing continuous perfusion with normal Tyrodes solution at 37^°^C containing (in mmol/L) NaCl (140), KCl (5), 1.2 MgCl_2_, glucose (5), HEPES (5), and CaCl_2_ (2) (pH adjusted to 7.4 with NaOH). To ensure that only nodal cells were included in the study, only those cells exhibiting spontaneous rhythmic beating were selected for recording. Experiments were performed using a Cs^+^-based pipette solution containing (in mmol/L): CsCl (130), NaCl (10), MgATP (1), Hepes 10, BAPTA (5), and amphotericin B (200 *μ*g/mL) (pH adjusted to 7.2 with KOH). The amphotericin-perforated patch technique was used to minimize dialysis of cytoplasmic constituents and generally reduce rundown (Yuill et al. 2010). Patch pipettes (Corning 7052 glass, AM Systems, Everett, MD) were pulled using a Model P2000 puller (Sutter Instruments, Novato, CA) and fire polished on a microforge (MF-83 Narishige) to resistances of ∼5 MΩ. Recordings were made using an Axopatch 200B amplifier. Data were digitized using a Digidata 1322A A/D interface and acquired using Clampex software (Molecular Devices, Sunnyvale, CA). Capacitative transient currents elicited by a 10 mV hyperpolarizing step were measured and compensated. The area under the transient was used to calculate cell membrane capacitance, enabling comparison of cell surface area of AVN myocytes from both control and STZ-treated animals.

### Drugs and chemicals

Aristar grade chemicals (BDH) dissolved in deionized water (18.4 MΩ) were used for myocyte isolation and pipette solutions, while Analar grade chemicals (BDH) were utilized for external solutions. All drugs were supplied by Sigma-Aldrich.

### Data acquisition and analysis

Voltage clamp protocols were generated using PClamp 8 (Molecular Devices). Data were digitized at 2 kHz and recorded online. Data were stored on computer, and analyzed using Clampfit (Molecular Devices), Excel (Microsoft, Redmond, WA) and Origin (Microcal, Northampton, MA) software. Numerical values in the text are shown as mean ± SEM and were compared statistically using the Student’s two-way *t*-test in Excel (Microsoft) and Sigmaplot (Systat, San Jose, CA) software. *P* values of <0.05 were considered as statistically significant.

The voltage-dependent activation of *I*_CaL_ was fitted using the Boltzmann equation:



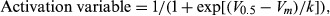
1

where *V*_*m*_ = test potential, *V*_0.5_ is the membrane potential exhibiting half-maximal activation, and *k* is the slope factor.

The steady-state, voltage-dependent inactivation of *I*_CaL_ was described by the equation:



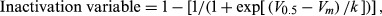
2

where *V*_*m*_ = the prepulse potential, *V*_0.5_ = the membrane potential at which inactivation was half-maximal and k describes the slope of the inactivation curve.

The time dependence of current inactivation was described by the following equation:



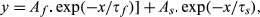
3

where *A*_*f*_ and *A*_*s*_ are the current components described by the time constants *τ_f_* and *τ_s_*.

The time course of recovery from inactivation of *I*_CaL_ was described by the equation:



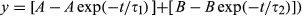
4

where y describes the fraction of recovery of *I*_CaL_ from inactivation at time *t*; A and B describe the data fitted by each exponential, and *τ*_1_ and *τ*_2_ are the time constants for each exponential.

## Results

### Cellular capacitance

**Figure 1:**
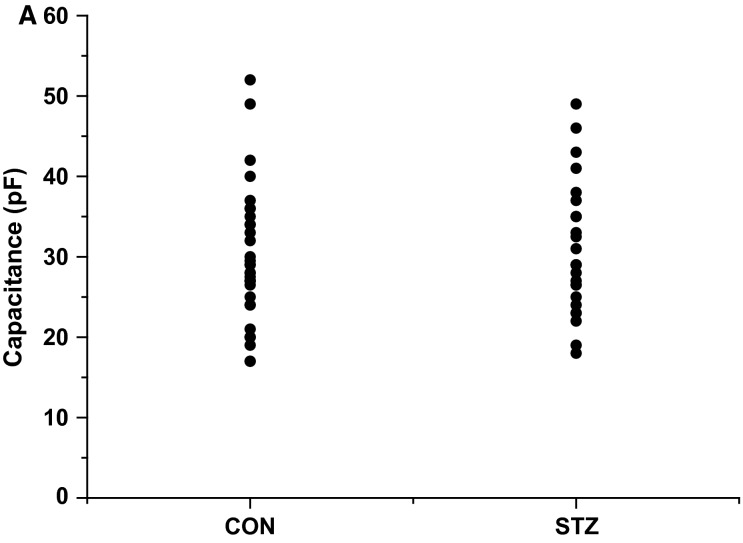
The capacitance of atrioventricular nodal (AVN) myocytes isolated from control and streptozotocin (STZ) rats. (A) Scatter plot showing the capacitance measurements for the population of control and STZ AVN myocytes. There was no significant difference in cell capacitance and therefore cell size between the two populations of cells (CON = 29.5 ± 1.6 pF, *n* = 35, STZ = 30.9 ± 1.4 pF, *n* = 29; *P* > 0.4).

The AVN isolation produces a heterogeneous population of cells (Meijler and Janse 1988), so to ensure the identity of the cells included in the study were nodal in origin, we only included cells that exhibited regular spontaneous electrical activity. The cells displayed the characteristic AVN morphology described in previous studies on rabbit, guinea pig, and rat AVN myocytes (Hancox et al. 1993; Yuill and Hancox 2002; Yuill et al. 2010). Cells from the two groups of animals appeared to be similar in size and shape, which is consistent with our previous study in rat AVN that explored the effects of short-term diabetes on AVN function (Yuill et al. 2010). To assess whether there were any differences in cell size between the two populations we measured the cell capacitance for all cells studied. Although there was some degree of variation in cell size within both groups of cells, there was no significant difference (*P *>* *0.4) between mean capacitance in the control group (29.5 ± 1.6 pF; *n* = 35) and the STZ (30.9 ± 1.4 pF; *n* = 29) (see Fig.1B).

### Diabetes mellitus reduces peak L-type calcium current

Using selective recording conditions to fully isolate *I*_CaL_, we examined the effects of long-term DM on *I*_CaL_ channel characteristics. Inward currents were elicited by sequential step depolarizations, from a holding potential of −40 mV, to more positive test potentials, for a duration of 500 ms. Application of nifedipine (1 *μ*mol/L), a selective L-type calcium channel blocker, abolished the inward current at all test potentials. After prolonged exposure to the diabetic state, *I*_CaL_ was clearly and significantly reduced in cells from STZ-treated rats compared to control.

**Figure 2:**
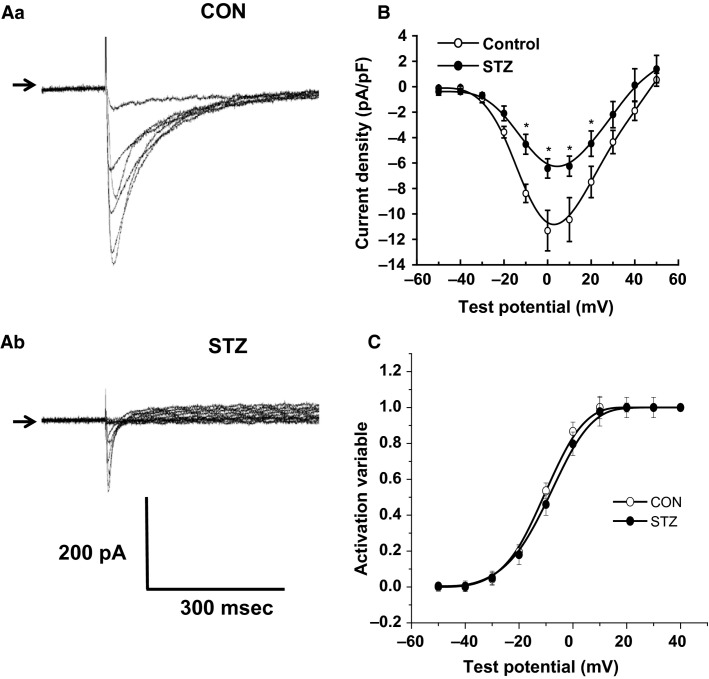
The mean current–voltage relationship for L-type calcium channel current in atrioventricular nodal (AVN) myocytes isolated from control and streptozotocin (STZ) rats. (Aa) shows families of currents from control AVN myocytes, elicited by incremental 500 ms step depolarizations, at a frequency of 0.2 Hz, from a holding potential of −50 mV, to more positive test potentials. (Ab) shows representative currents elicited in STZ AVN myocytes. (B) shows the mean current–voltage (I–V) relation for *I*_CaL_ in AVN mycoytes from control (open circles) and STZ rats (closed circles, *n* ≥ 12). For each cell, the amplitude of *I*_CaL_, at different voltages was normalized to cell capacitance, and plotted as a function of voltage. The mean amplitude of *I*_CaL_ was significantly smaller in STZ compared to control cells. Asterisks indicate statistical significance (*P* < 0.05). (C) The mean steady-state activation curve for *I*_CaL_ in control and STZ cells. In control cells, the mean value of *V*_0.5_ was −10.9 ± 1.8 mV with a slope factor of 11.1 ± 0.9 mV, while in STZ cells, the *V*_0.5_ = 9.4 ± 1.4 mV with a slope factor of 10.3 ± 1.2 mV (*P* < 0.8, *n* ≥ 12). Therefore, the amplitude of *I*_CaL_ was significantly reduced in STZ compared to control, without altering the voltage dependence of *I*_CaL_ activation.

Figure2A shows representative families of currents recorded in control (Fig. 2Aa) and STZ cells (Fig 2Ab). The currents recorded in the cell from the STZ rat were substantially smaller than those seen in the age-matched control. We constructed current–voltage (I–V) relationships by plotting the peak current elicited at each test potential normalized to cell capacitance (current density), against membrane potential, and the mean values (*n* ≥ 12) are shown in Figure2B. Peak *I*_CaL_ occurred at 0 mV and this was reduced by 43% in STZ cells compared to control cells (*P *<* *0.01). Figure2B shows the mean I–V relationships in control and STZ cells, where a significantly smaller *I*_CaL_ was observed across a range of test potentials, with significance (*P ≤* *0.05) observed at all potentials between −10 and +20 mV. Steady state activation curves using equation 1, were plotted to examine whether there was any significant effect on activation of the current. The activation curves from the pooled data are shown in Figure2C. In control cells, the half-activation potential was −10.9 ± 1.8 mV, with a slope factor, *k*, of 11.1 ± 0.9 mV. This was not significantly different from the STZ cells, where the half-maximal activation was −9.4 ± 1.4 mV, and the slope factor was 10.3 ± 1.2 mV (*P* < 0.8, *n* ≥ 12). Thus, the data show that in rats with STZ-induced DM, peak *I*_CaL_ density was significantly reduced. However, there was no significant effect on the voltage dependence of activation.

#### L-type calcium current voltage-dependent inactivation is modified by diabetes mellitus

**Figure 3:**
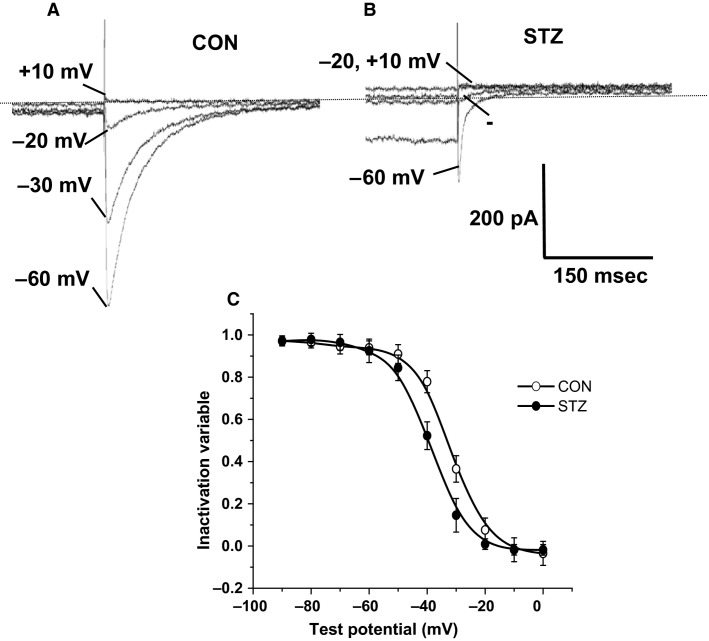
The voltage dependence of *I*_CaL_ inactivation in control and streptozotocin (STZ) atrioventricular nodal (AVN) myocytes. (A) Representative current records obtained from control AVN myocytes during a 300 ms duration voltage command to 0 mV, following conditioning 1000 ms prepulses to the potentials indicated. Currents were elicited at a frequency of 0.1 Hz. (B) Family of current records from STZ AVN myocytes, using the same protocol as described above. (C) The voltage dependence of *I*_CaL_ inactivation. There was a significant hyperpolarizing shift in the voltage dependence of inactivation in STZ cells, compared to control (CON, *V*_0.5_, −32.3 ± 1.4 mV, *k *=* *7.9 ± 0.8 mV, STZ, *V*_0.5_ = −39.6 ± 2.1 mV, *k *=* *8.3 ± 0.6 mV, *P* < 0.01, *n* ≥ 10).

The steady-state inactivation parameters for *I*_CaL_ were then determined by delivering a series of prepulses (1000 ms duration) applied at a frequency of 0.1 Hz, incrementing (10 mV steps) from −90 mV to +20 mV from a holding potential of −80 mV. The conditioning pulses were followed by a test pulse to 0 mV for 500 ms. Families of selected test currents recorded in cells from control and STZ-treated animals are shown in Figures3A and B. Mean data (Fig.3C; *n* ≥ 10) were plotted as peak current measured during the test potential for each prepulse potential, and were fitted to the Boltzmann equation (2). Figure3C shows a significant (*P *<* *0.01) hyperpolarizing shift in the half-inactivation potential in cells from STZ-treated rats (*V*_0.5_ = −39.6 ± 2.1 mV, *k *=* *8.3 ± 0.6 mV), compared to age-matched controls (*V*_0.5_, −32.3 ± 1.4 mV, *k *=* *7.9 ± 0.8 mV).

### Diabetes mellitus alters the time dependence of L-type calcium current inactivation

**Figure 4:**
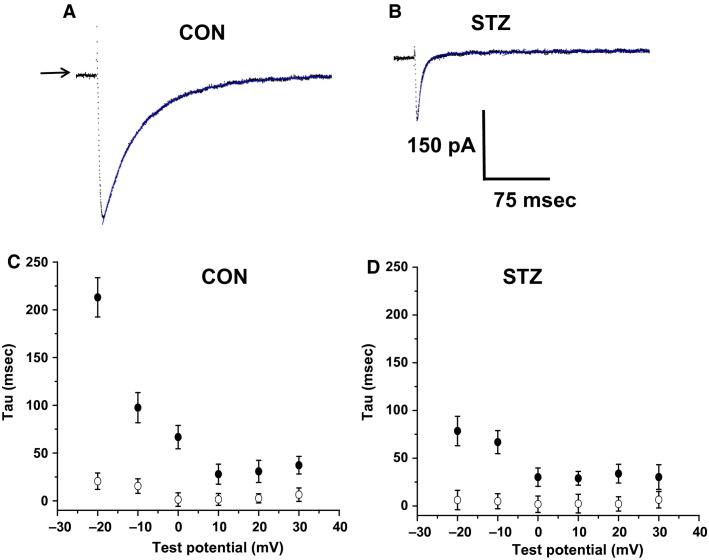
The time course of *I*_CaL_ inactivation in control and streptozotocin (STZ) atrioventricular nodal (AVN) myocytes. (A). Representative current trace (dotted line) elicited by a step depolarization from −40 mV to +0 mV for 500 ms duration in a control cell. The current decay was fitted with a double exponential function, which overlays the current trace (solid line, blue). (B) Representative current trace elicited using the same protocol as above, in an STZ AVN myocyte. (C) Mean data for control cells showing the fast (*τ*f) and slow (*τ*s) time constants describing inactivation, over a range of test potentials (−20 to +30 mV). Open circles depict *τ*f, closed circles depict *τ*s. (D) Mean data for *τ*f and *τ*s describing *I*_CaL_ inactivation in STZ AVN myocytes, over a range of test potentials (−20 to +30 mV). Open circles depict *τ*f, closed circles depict *τ*s. There was a significant difference between *τ*f and *τ*s in control and STZ myocytes at −20, −10, and 0 mV; *P* < 0.05, *n* ≥ 8).

Examination of the traces illustrated in Figures2 and 3 suggest differences in the kinetic properties displayed by the families of *I*_CaL_ in control compared to STZ cells. To explore this further, the time dependence of inactivation of *I*_CaL_ was compared in the two groups. The kinetics of *I*_CaL_ have been demonstrated previously to be described by a biexponential function, where a fast component of the current (*A*_*f*_) can be described by the time constant (*T*_*f*_), and a slow current component (*A*_*s*_) can be described by a time constant (*T*_*s*_) (see equation 3). Figure4 shows representative currents elicited by a step depolarization from −40 mV to 0 mV in a control cell (Fig.4A) and a cell from an STZ animal (Fig.4B). The current decay was fitted with a biexponential function (illustrated by the solid line) to determine the inactivation time constants across a range (−20 to +30 mV) of test potentials. Figures4C and D show the results of this analysis pooled from eight cells in control animals and 10 from STZ rats. The values for *T*_*f*_ were 5–10-fold smaller than the values for *T*_*s*_. Both exhibited voltage dependence and became increasingly smaller at more positive test potentials in both age-matched control cells and STZ cells. The amount of current described by each function did not differ significantly over the range of test potentials. However, significant differences in the time constants of inactivation were observed between cells from control and STZ-treated animals, with the latter exhibiting a significant reduction in the slow decay constant, *T*_*s*_, at test potentials between −20 and 0 mV (*P* < 0.05). These data demonstrate that in addition to a reduction in peak amplitude of *I*_CaL_, AVN cells from animals with DM exhibit a much more rapid decay overall.

### Diabetes mellitus slows the restitution properties of L-type calcium current

**Figure 5:**
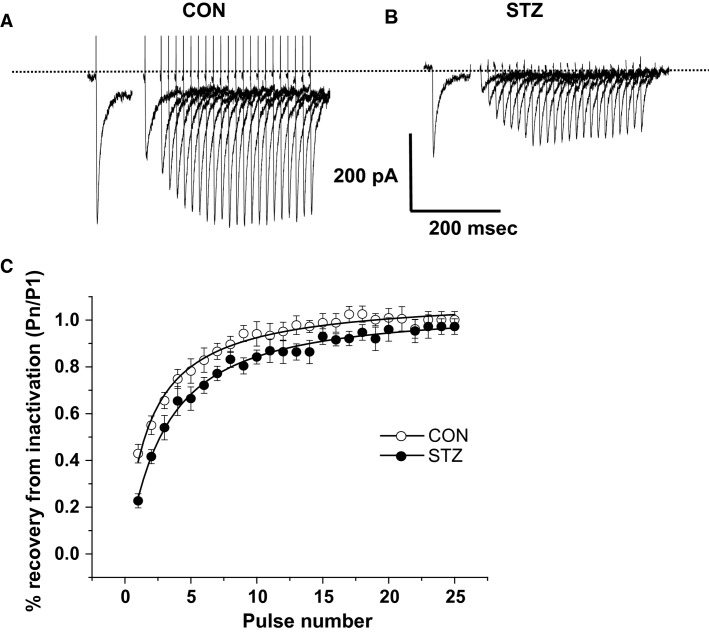
The restitution of *I*_CaL_ in control and streptozotocin (STZ) atrioventricular nodal (AVN) myocytes. Representative *I*_CaL_ traces elicited using a double pulse protocol (P1, P2), from a holding potential of −50 mV in control AVN myocytes. The interval between P1 and P2 was increased incrementally, by 50 ms at a frequency of 0.1 Hz. (A) Representative *I*_CaL_ traces elicited using the same protocol as above, in STZ AVN myocytes. (B) Plot showing the recovery from inactivation. For each P1–P2 interval, the magnitude of peak *I*_CaL_ during P2 was expressed as a proportion of P1. Recovery of inactivation was significantly slower in STZ AVN myocytes, compared to control cells (*P* < 0.05, *n* = ≥8).

The prior experiments established that DM is associated with changes in the time-dependent and voltage-dependent properties of *I*_CaL_ inactivation. To complete the analysis, we then studied the effects of DM on recovery from inactivation. Restitution of *I*_CaL_ follows a biexponential time course and consists of an initial fast phase (*T*_1_) succeeded by a slower phase (*T*_2_). We measured restitution using a double-pulse protocol, where currents were elicited by an initial step depolarization (P1) from −40 to 0 mV, and subsequent step depolarizations (Pn) elicited with increasing intervals from P1 in variable incremental intervals. Representative current traces for control and an STZ cell are shown in Figures5A and B, respectively. The recovery from inactivation was calculated from the current amplitude for each incrementing interval, as a fraction of the initial peak current (Pn/P1), and was then plotted against pulse number. The pooled data are shown in Figure5C. Analysis shows that there was a significant delay in the recovery of inactivation in STZ cells compared to control (*P* < 0.05). When the mean data were fitted with a biexponential function (Equation 4), the time constants in control cells (*T*_1_ = 10.4 ± 0.9 ms, *T*_2_ = 44.3 ± 1.8 ms) were found to be significantly (*P* < 0.05) faster than those for DM-affected cells in STZ rats (*T*_1_ = 20.1 ± 1.1 ms, *T*_2_ = 149.2 ± 2.4 ms *P* < 0.05).

## Discussion

To the best of our knowledge, this study is only the second to have investigated the effects of diabetes mellitus on rat AV node single-cell electrophysiology. The previous study in rat AVN cells was performed 14–21 days after induction of diabetes while this study was performed after 12 weeks. These two studies might reflect short-term and long-term SZT-induced diabetes. We have previously demonstrated that DM can alter membrane excitability in rat AVN myocytes, reducing action potential velocity and upstroke characteristics (Yuill et al. 2010). Here, we demonstrate that that *I*_CaL_ is downregulated in the AVN from rats with STZ-induced type 1 DM, and that this is likely to underlie the changes in the profile of spontaneous action potentials seen in diabetic AVN cells.

### L-type calcium current in rat atrioventricular node cells

Much of what is currently known about the properties of AVN cells is derived from rabbit tissue, and this has been comprehensively reviewed previously (Hancox et al. 2003). Rat AVN has been shown to exhibit the same structural characteristics as rabbit AVN (Stephenson et al. 2012). While there is limited information regarding AVN cellular characteristics available from other animal models, such as guinea pig (Yuill and Hancox 2002) and mouse (Mangoni et al. 2006; Nikmaram et al. 2008; Marger et al. 2011), our previous study in rat (Yuill et al. 2010), reported that the action potential profile and zero current potential, were comparable to prior findings in rabbit AVN (Hancox et al. 1993; Munk et al. 1996; Hancox and Mitcheson 1997; Cheng et al. 2009). However, to date, there has been no detailed characterization, of the ion channel characteristics of rat AVN.

The generation of normal cardiac rhythm and propagation through the AVN, is dependent on the presence of voltage-gated L-type calcium channels, which are heteromeric complexes comprising four subunits (*α*1, *β*, *α*2/*δ*), and the *α*1 subunit exists in multiple isoforms. Typically, the two isoforms *α*1C (Cav1.2) and *α*1D (Cav 1.3) are expressed in the heart, with Cav1.3 being expressed predominantly in the SAN and AVN regions (Mangoni et al. 2003; Matthes et al. 2004). Cav1.3 has subtle differences in its kinetic profile, and is activated at more negative membrane potentials than Cav1.2, allowing it to contribute to diastolic depolarization (Takimoto et al. 1997).

Here, we have shown the peak *I*_CaL_ to be at 0 mV, and half-maximal activation in control cells to be −10.9 mV, which is comparable to previous findings in rabbit (Cheng et al. 2009). However, previous studies in rabbit AVN have shown activation parameters to be ∼10 mV more depolarized (Hancox et al. 1993; Cheng et al. 2009). Mouse AVN has been demonstrated to express greater levels of Cav1.3 than Cav1.2, and *I*_CaL_ exhibited peak current around −10 mV, with a more hyperpolarized activation profile (Marger et al. 2011).

However, the voltage dependence of *I*_CaL_ inactivation parameters that we observed in rat AVN myocytes, were very similar to previous studies in rabbit (Matthes et al. 2004), *V*_0.5_ = −25 mV; *V*_0.5_ = −29.4 mV). We also found that the recovery from inactivation exhibited a biexponential time course, but was significantly faster than previous studies in rabbit (Workman et al. 1999; Cheng et al. 2009). The time-course of current decay exhibited a fast and slow component that were voltage dependent, and these were also similar values to those observed in rabbit AVN (Hancox and Levi 1994), but were significantly faster than AVN myocytes with abnormal morphology (Nakayama et al. 1984). Previously, nonselective recording conditions revealed that L-type Ca^2+^ current was reduced and there appeared not to be a shift in the voltage dependence of activation (Yuill et al. 2010) findings that are consistent with this study where L-type Ca^2+^ current was recorded under more selective conditions. There are clearly subtle differences in the *I*_CaL_ channel characteristics reported in AVN myocytes of different species. This could reflect the variation in recording parameters within different laboratories and the heterogeneity of the AVN region used within each study. However, these changes in profile may well be genuine physiological differences and reflect species differences in expression levels of Cav1.3 and Cav1.2 isoforms in AVN myocytes (Zhang et al. 2011). While there are differences between studies, overall, the properties of *I*_CaL_ activation, inactivation, restitution, and the time dependence of inactivation are largely comparable between rat and rabbit, suggesting that the rat model of AVN has utility for investigation of cellular electrical function of the region.

### Modulation of L-type calcium current by diabetes mellitus

Changes in action potential profile are generated by alterations in sarcolemmal ionic fluxes, and previous studies have indicated that induction of diabetes can modulate a variety of cardiac ion channels, creating a significant impact on cardiac function. Available studies of the effects DM on L-type calcium channel activity in the literature, are largely focused on rat ventricle, and demonstrate a diversity of different findings. This variation may derive from differences in experimental procedures, and implementation and duration of the diabetic state. Some groups suggest that induction of DM has no significant effect on *I*_CaL_ amplitude (Choi et al. 2002; Ding et al. 2006) or voltage-dependent activation, inactivation and time-dependent current properties (Arikawa et al. 2002). However, these studies were conducted at room temperature, and this has been shown to profoundly influence L-type calcium channel properties, particularly with respect to diabetes (Pabbathi et al. 2004), and as such inferences drawn from data that was obtained under nonphysiological recording conditions may be questionable. Other studies have also observed no difference in *I*_CaL_ amplitude after the induction of DM (Lacombe et al. 2007), and while these experiments were performed at 35°C, they were conducted simultaneously with intracellular calcium measurements, and thus the chelation of intracellular calcium may have influenced the electrophysiological findings by altering calcium-dependent inactivation parameters. Conversely, others have demonstrated an augmentation of *I*_CaL_ amplitude in diabetic ventricular myocytes compared to control cells (Jourdon and Feuvray 1993). These experiments were also performed at room temperature, and the ventricular myocytes exhibited vastly depolarized resting membrane potentials (−66 mV), indicating the likelihood of cellular damage resulting from the isolation procedure, and perhaps negating the findings in this study.

There is more general consensus that diabetes inhibits *I*_CaL_ amplitude, both in STZ-induced diabetes (Wang et al. 1995; Chattou et al. 1999; Bracken et al. 2006), which is in keeping with our current findings in STZ rat AVN. Previous studies in an insulin-deficient diabetic mice model (Akita mouse) have shown that the peak amplitude of L-type calcium current is reduced compared to control (Lu et al. 2007). Studies of type II diabetes in mouse (db/db; [Pereira et al. 2006; ] and rat (Zucker diabetic fatty; (Howarth et al. 2011), have also demonstrated a significant decrease in *I*_CaL_ amplitude.

While these studies agree that *I*_CaL_ amplitude is reduced, the effects of diabetes on *I*_CaL_ gating characteristics are varied. We saw no change in the activation parameters, which contrasts with findings of an observed rightward shift in the activation variable (Lu et al. 2007). Conversely, the same authors also observed a depolarizing shift in the inactivation of the channel, while in our study, the voltage dependence of inactivation was shifted in the hyperpolarizing direction, suggesting that at physiological voltages, there would be less steady-state current available compared to control. The leftward shift of inactivation, faster inactivation time course, and slower restitution all point to stabilization of the inactivated channel state in DM. The combination of reduced L-type Ca^2+^ current amplitude and altered inactivation/current restitution might be anticipated both to slow AV nodal conduction and delay AV nodal action potential restitution. One of the most striking findings in this study was the difference in the time dependence of current decay between the control and STZ cells. We observed a significant reduction in the fast and slow time constants of current inactivation compared to control. While previous studies have observed changes in the kinetics of *I*_CaL_ decay following induction of diabetes, the time course of current decay has been shown to increase (Chattou et al. 1999; Bracken et al. 2006; Lu et al. 2007). It is likely that this difference in channel kinetics may relate to different levels of Cav1.2 and Cav1.3 channel expression between the AVN region and the ventricle, which may be further modified by diabetes.

Furthermore, previous studies have identified that intracellular calcium dynamics are greatly altered in diabetic ventricular myocytes, with a reduction in calcium transient amplitude, a prolonged time course of the calcium transient, depressed calcium loading of the sarcoplasmic reticulum, leading to an increase in basal calcium concentrations (Yaras et al. 2005; Pereira et al. 2006). We postulate that in these conditions, an increase in calcium concentration could facilitate calcium-dependent calcium current inactivation. It is possible therefore that the decrease in the time constants of current inactivation that we observed in the STZ myocytes, may have resulted from modulation of the calcium-dependent component of current inactivation. This is of particular importance, as intracellular calcium homeostasis in nodal myocytes (SAN and AVN) has been shown to contribute to spontaneous electrical activity and automaticity, in concert with sarcolemmal ionic fluxes (Ridley et al. 2008). Any changes in calcium dynamics induced by diabetes could therefore influence action potential generation, fundamentally altering the physiology of the cardiac conduction system. There is, however, little information available in the literature concerning measurement of global and local calcium in diabetic SAN and AVN myocytes, which will be of key importance for future understanding of the effects of diabetes on cardiac function. Our results demonstrate that *I*_CaL_ activity is downregulated in AVN myocytes from STZ rats, which is in keeping with previous studies in rat ventricle. While we have not specifically investigated the mechanisms that underlie these findings, we speculate that there are changes in both channel trafficking and modulation of channel activity by regulatory proteins, based on previous work (Lu et al. 2005), which demonstrated that depression of *I*_CaL_ activity coincided with a reduction in PI3 kinase signaling. Further studies indicated that in type I diabetic Akita mice, the reduction in *I*_CaL_ amplitude compared to controls, proceeded from impaired channel trafficking to the cell membrane mediated via the PI3 kinase signaling pathway (Lu et al. 2007). However, changes in the channel activation and inactivation kinetics were independent of the PI3 kinase signaling pathway, suggesting that multiple mechanisms are involved in regulation of *I*_CaL_ by diabetes. Given the changes in both channel amplitude and gating kinetics in our present study, we speculate that similar processes may be responsible for altered *I*_CaL_ in rat AVN myocytes from STZ rats.

### Clinical significance and conclusions

Atrioventricular nodal dysfunction can play a vital role in a variety of cardiac arrhythmias. *I*_CaL_ is responsible for the action potential upstroke in AVN myocytes, and is therefore fundamental to conduction through the region. Depression of *I*_CaL_ activity via reduced trafficking to the membrane, or altered channel kinetics, will decrease AVN conduction velocity, and can therefore induce ECG changes, including first to third degree AV block (Watanabe 1981; Watanabe et al. 2012). There is also evidence to suggest that AV block can initiate the development of QT prolongation and of Torsade de pointes (Tsuji et al. 2002), which can result in sudden cardiac death. The reduction in *I*_CaL_ seen in this study in AVN myocytes following induction of diabetes is consistent with abnormal AVN function and could contribute to arrhythmogenic cardiac activity. Arrhythmias are a major cause of mortality in patients with diabetes (Nakou et al. 2012), and it is therefore imperative that the underlying cellular pathophysiology is investigated in order to elucidate the causes and potential therapeutic strategies, of cardiac electrical dysfunction in diabetes.
